# Shark Fin Electrocardiogram: A Deadly Electrocardiogram Pattern in ST-Elevation Myocardial Infarction (STEMI)

**DOI:** 10.7759/cureus.15989

**Published:** 2021-06-28

**Authors:** Amit K Jaiswal, Sunil Shah

**Affiliations:** 1 Internal Medicine, Ministry of Health, Male', MDV; 2 Medicine, Ministry of Health, Male', MDV; 3 Internal medicine, California Institute of Behavioral Neurosciences & Psychology, California, USA

**Keywords:** shark fin, lambda wave, giant r wave, triangular qrs-st-t waveform, stemi

## Abstract

Shark fin electrocardiographic (ECG) pattern, also known as 'Lambda-wave', 'giant R waves', or 'triangular QRS-ST-T waveform' is a dangerous ECG pattern associated with ST-elevation myocardial infarction (STEMI). It is formed by the fusion of QRS, ST, and T waves and predicts the high risk of mortality due to cardiogenic shock and ventricular fibrillation. The management should be aggressive with reperfusion via thrombolysis or percutaneous intervention, ideally in the intensive care unit with ventricular assist devices. This ECG pattern may be misdiagnosed as wide complex tachycardia or the ECG changes of hyperkalemia. Thus, differentiating it from other conditions causing similar ECG changes and prompt management is highly important to save the patient from serious complications. Here we have presented a case of STEMI with shark fin ECG associated with pulmonary edema (Killip class III acute myocardial infarction).

## Introduction

Shark Fin pattern is an uncommon but high-risk electrocardiographic (ECG) pattern formed by fusion of QRS, ST-segment, and T waves [1]. Myocardial infarction with shark fin ECG pattern most commonly involves occlusion of the left main coronary artery and is associated with a high risk of death due to cardiac arrest and cardiogenic shock [2]. Therefore, aggressive management is needed in such patients. It can be easily mistaken for other conditions such as a wide complex tachycardia and hyperkalemia-associated changes in ECG. Early recognition and differentiating it from other causes of similar changes in ECG is important to prevent higher rates of acute complications such as cardiogenic shock and ventricular arrhythmia.

## Case presentation

A 48-year-old male presented to the emergency department (ED) with complaints of chest pain, breathing difficulty, and vomiting for two to three hours. He also complained of sweating and orthopnea. On examination, his blood pressure was 130/70 mmHg, pulse rate 120 beats/minute, respiratory rate 24 cycles/min, temperature 37-degree centigrade, and oxygen saturation 86% in room air. There were coarse crepitations on both the lungs. He had a history of hypertension and hyperlipidemia under irregular medication due to poor adherence to treatment. He had a 20-pack-year smoking history, but he denied intake of alcohol and illicit drug use.

**Figure 1:**
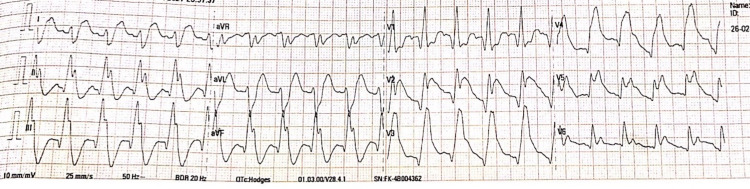
ECG at Presentation

He was placed in a reclined position at a 45-degree angle. Oxygen was given at the rate of 6 liters/minute to maintain oxygen saturation of more than 96%. The ECG done at the emergency department (ED) is shown in Figure 1.

**Figure 2:**
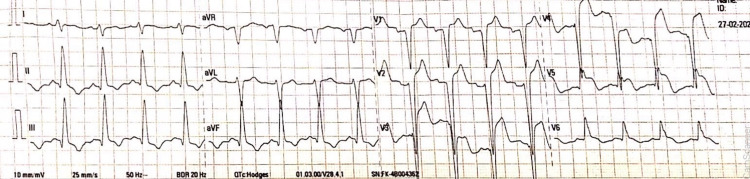
ECG After 90 Minutes of Thrombolysis

A comprehensive metabolic panel along with serum potassium level was within normal limits. The diagnosis of extensive ST-elevation myocardial infarction (STEMI) was made. Aspirin 300 mg and atorvastatin 80 mg were given immediately. Due to the unavailability of recombinant tissue plasminogen activator (rTPA), streptokinase was given for thrombolysis. The ECG at 90 minutes post thrombolysis is as shown in Figure 2.

The patient had decreased oxygen saturation (< 90%) even after 2 mg of morphine three times and 140 mg of furosemide. Continuous positive airway pressure (CPAP) was then administered to maintain saturation. Once he was resuscitated, he was sent to the higher center for percutaneous coronary intervention (PCI). In the higher center, coronary angiography was done, and a critical occlusion of the left anterior descending coronary artery was relieved with thrombectomy and stent placement. His condition was stable post this.

## Discussion

Shark Fin ECG (SFE) pattern is a high-risk giant wave (amplitude ≥1 mV) ECG pattern associated with a large burden of myocardial ischemia [3]. It is also known as 'Lambda-wave', 'giant R waves', or 'triangular QRS-ST-T waveform' [4]. This ECG pattern consists of blurring of the QRS complex due to its fusion with the ST-segment and the T-wave showing a triangular lambda pattern where a positive deflection in the leads suggests ischemia in corresponding areas [1]. According to one study done by Cipriani et al. [3], only 1.4% of patients admitted due to STEMI developed this ECG pattern, illustrating its rarity.

SFE is usually associated with the occlusion/sub-occlusion of the left main coronary artery, left anterior descending artery, or proximal right coronary artery, especially in the absence of collateral coronary circulation [3]. The electrogenesis of this wave in patients with acute myocardial infarction (AMI) remains to be elucidated. The expansion of the left ventricular cavity during acute myocardial infarction (Brody’s hypothesis), the increase in tissue resistance affecting myocardial conductivity factor (“the solid angle theorem”), or the difference in response of epicardial and endocardial action potentials are some of the hypotheses for the genesis of this waveform [5-7].

The SFE associated AMI is more frequently complicated by cardiogenic shock or cardiac arrest; however, our case was presented with pulmonary edema (Killip class III). Killip classification of acute myocardial infarction is as shown in Table 1 [8].

**Table 1:** Killip Classification of Acute Myocardial Infarction SBP= Systolic Blood Pressure

Killip Class	Characteristic Features
Class I	No clinical evidence of heart failure
Class II	Findings consistent with mild to moderate heart failure (third heart sound S3, lung rales, or jugular venous distension)
Class III	Pulmonary edema
Class IV	Cardiogenic shock or arterial hypotension (SBP<90 mmHg)

This ECG finding can be confused with wide complex tachycardia, ECG changes in myopericarditis, and hyperkalemia. Furthermore, it may be confused with the tombstoning ECG pattern of STEMI seen in 10-15% of the patients. The features of tombstoning ECG differ from those of Shark Fin pattern regarding the presence of an elevated, convex upward ST-segment that connects a small and short R wave with a higher and broader T wave [9].

The management of the AMI with SFE should be prompt and more aggressive in an intensive care unit equipped with ventricular assist devices to address the impending cardiogenic shock and increase the survival rate. With both electrical and hemodynamic instability, this ECG pattern is associated with poor prognosis in patients with STEMI. Most of them experience ventricular fibrillation and/or cardiogenic shock [3]. The increased frequency of development of ventricular fibrillation, especially in the acute phase of these patients, causes a high risk of mortality [3].

## Conclusions

The shark fin waveform is an uncommon but high-risk ECG pattern of STEMI which should be diagnosed early and differentiated from other conditions causing similar waveforms such as wide-complex tachycardia and hyperkalemia. The shark fin STEMI is associated with a high risk of both ventricular fibrillation (VF) and cardiogenic shock, accounting for increased in-hospital mortality. Thus, it requires aggressive management, including mechanical circulatory support.
